# Detection of blueberry stunt phytoplasma in Eastern Canada using *cpn60*-based molecular diagnostic assays

**DOI:** 10.1038/s41598-021-01439-4

**Published:** 2021-11-11

**Authors:** Christine Hammond, Edel Pérez-López, Jennifer Town, Charles Vincent, Debra Moreau, Tim Dumonceaux

**Affiliations:** 1 Agriculture and Agri-Food Canada, Saskatoon, SK Canada; 2 Département de Phytologie, Université Laval, Faculté des Sciences de l’Agriculture et de l’Alimentation, Québec, QC Canada; 3 Agriculture and Agri-Food Canada, Saint-Jean-sur-Richelieu, QC Canada; 4 Agriculture and Agri-Food Canada, Kentville, NS Canada

**Keywords:** Plant sciences, Bacterial techniques and applications

## Abstract

Blueberry stunt phytoplasma (BBSP; ‘*Candidatus* Phytoplasma asteris’) is an insect-vectored plant pathogen that causes severe yield losses in blueberry (*Vaccinium corymbosum*), which is the most valuable fruit crop in Canada. Rapid, field-based diagnostic assays are desirable tools for the control of BBSP, as part of an integrated, proactive approach to production management termed biovigilance. We designed and validated a chaperonin-60 (*cpn60*)-targeted LAMP assay for detection of BBSP, providing a rapid, low cost, field-deployable diagnostic option. Our validation demonstrates that the assay is reproducible, with high analytical specificity and improved sensitivity when compared with 16S rRNA nested PCR. We applied the validated LAMP assay to nearly 2000 blueberry samples from Québec and Nova Scotia over three growing seasons (2016–2018). Our surveys revealed that BBSP is present in most sites across both provinces, though detection of the pathogen in individual plants varied in different tissues across sampling dates and across years, and evidence of spread between plants was limited. To quantify pathogen load in select plants, we designed additional qPCR and ddPCR assays, also based on *cpn60*. We found that pathogen load fluctuates in individual plants, both within and between growing seasons. Finally, we designed an interactive map to visualize the results of our surveys. These results provide a validated diagnostic assay that can be used as part of a biovigilance strategy for detecting and controlling infections caused by BBSP.

## Introduction

In Canada, two species of blueberry that are native to North America are commercially exploited: the northern highbush blueberry (*Vaccinium corymbosum* L. Ericaceae), widely cultivated in British Columbia, Ontario, Québec and Nova Scotia, and the lowbush blueberry (*Vaccinium angustifolium* Aiton and *Vaccinium myrtilloides* Michx.), found mainly in Québec, Nova Scotia and New Brunswick^1^. In 2018, blueberries were the cultivated fruit with the highest farm gate and export value in Canada, accounting for > 50% of the total fruit value exported (total of highbush and lowbush blueberries)^2^.

Blueberry stunt is a disease caused by a phytoplasma, principally ‘*Candidatus* Phytoplasma asteris’, which is vectored primarily by the sharp-nosed leafhopper *Scaphytopius magdenalsis*^3,4^ though transmission has also been observed by *S. acutus* and *S. frontalis*^5^. As reviewed by Ramsdell et al.^6^, the disease was first reported in New Jersey in 1942 and substantial negative impacts on blueberry quality and production have been attributed to BBSP^7^. Symptomatically, BBSP causes internodes to be shortened, leading infected bushes to appear severely stunted with bushy branches. Leaves are cupped slightly downward and may also have chlorotic edges that turn red late in the growing season. Fruit on infected plants ripens late or not at all. Often, infected bushes go undetected because symptoms can be subtle, especially early in the disease, or easily mistaken for other diseases. Furthermore, surveys of blueberry farms in Québec have revealed plants co-infected with BBSP phytoplasma and viruses^8^, complicating symptomatic detection. Knowledge gaps remain regarding the spread of the disease within individual plants, between plants at the field scale and at the wider geographical scale^9,10^.

Protecting crops such as blueberries from the potentially devastating effects of plant diseases requires an integrated and proactive approach. The concept of biovigilance describes an interlinked and interdisciplinary set of tools and research methods aimed at providing timely and accurate information to detect and anticipate the effects of new pests/diseases before they become a problem^11^. Monitoring diseases and their vectors is an essential part of blueberry integrated pest management, and diagnostic tests are needed to inform producers of disease status for targeted removal of infected plants as well as for optimal timing of management inputs^10^. In addition to the phenotypic changes associated with BBSP detection, phytoplasmas are unculturable, and therefore molecular approaches are necessary for identification and classification of the disease. Currently, the most common molecular method for identifying phytoplasma is through nested PCR amplification of the 16S rRNA-encoding locus^12^. Phytoplasmas are further classified into groups and subgroups according to RFLP analysis of their 16S rRNA-encoding loci, with more than 35 16Sr groups described to date^13^. Of those, members of the groups 16SrI, 16SrXIX, and 16SrXIII have been reported to affect blueberries in North America^14,15^. Although the 16S rRNA-encoding locus is regarded as the ‘gold standard’ for phylogenetic characterization of phytoplasmas, there are shortcomings to the use of this target. These include limited resolution of related taxa^16^ and 16S rRNA heterogeneity, which refers to the presence of two distinct copies of the gene found in the phytoplasma genome. The presence of two copies of the 16S rRNA-encoding gene that each provide different results can confound RFLP typing^17^. Furthermore, compared to alternative methods, such as loop-mediated isothermal DNA amplification (LAMP)^18,19^ and quantitative PCR (qPCR)^20^, nested PCR requires a longer processing time and the results are not quantitative.

Alternative targets to 16S rRNA-encoding genes are suitable for detection and typing of phytoplasmas, including ribosomal protein operon (*rp*)^21^ and chaperonin 60 (*cpn60*/groEL)^22^, which are protein-encoding gene sequences. A fragment of the *cpn60* gene sequence named the universal target (*cpn60* UT)^23^, has been suggested as a molecular barcode for the domain Bacteria^24^ and it has also been adapted and widely used to identify and characterize phytoplasmas^25–27^. An online database has become available for typing of phytoplasmas by *cpn60*^28^.

Recently, we examined BBSP-infected blueberries from Québec and Nova Scotia, finding two distinct 16S rRNA-encoding genes, a single *cpn60* sequence and a single *rp* sequence^27^, confirming 16S rRNA gene heterogeneity in this strain. These findings, along with the need for producers to have access to rapid, in-field diagnostics as part of a biovigilance-based disease management approach, motivated us to develop a rapid diagnostic assay for BBSP without the confounding effects of 16S gene heterogeneity.

LAMP is a highly specific method that uses four or six primers and a strand-displacement DNA polymerase to very rapidly amplify DNA at a single temperature. The isothermal aspect of the assay means that it can much more easily be deployed to a field setting than a PCR that requires temperature cycling. The assay is less costly and far less time consuming compared with nested PCR.

Our objective in this study was to develop new molecular methods to study the presence and distribution of the previously characterized BBSP in some blueberry fields in production in Eastern Canada, including determining the distribution of the phytoplasma within plant tissues, and over multiple growing seasons. Due to the volume of samples, a fast, convenient, inexpensive, and ideally quantitative assay was required to achieve these aims. We therefore designed *cpn60*-based LAMP and qPCR assays for rapid detection and quantification of BBSP in blueberry and conducted a thorough validation of the assays according to the standards set out by Burd^29^, including analytical sensitivity, analytical specificity, precision and comparison-of-methods. We then applied the validated LAMP assay to nearly 2000 blueberry samples from Québec and Nova Scotia over three field seasons to evaluate the incidence and spread of BBSP. Moreover, we have provided a visual interactive mapping application displaying time-course data for all plants sampled in this study. Finally, we applied the qPCR assay to selected samples to examine the changes in BBSP pathogen load in various tissues across time, providing data informing the optimal time and tissue type to sample for effective detection and management of BBSP.

## Results

### Quantitative PCR assay based on BBSP *cpn*60

The previously reported BBSP *cpn*60 UT sequence (GenBank KU523402)^27^ was used to design PCR primers and a hydrolysis probe for BBSP detection and quantification in blueberry tissues. To examine the PCR efficiency of this primer/probe set, a serial tenfold dilution series of BBSP *cpn60* UT-containing plasmid DNA was quantified using real-time PCR and the quantification cycle (C_q_) plotted against input template amount (Supplementary Fig. S1). The PCR efficiency (E) was determined to be > 1.99, indicating a highly efficient PCR. No amplification was observed from asymptomatic blueberry plant tissue.

**Figure 1:**
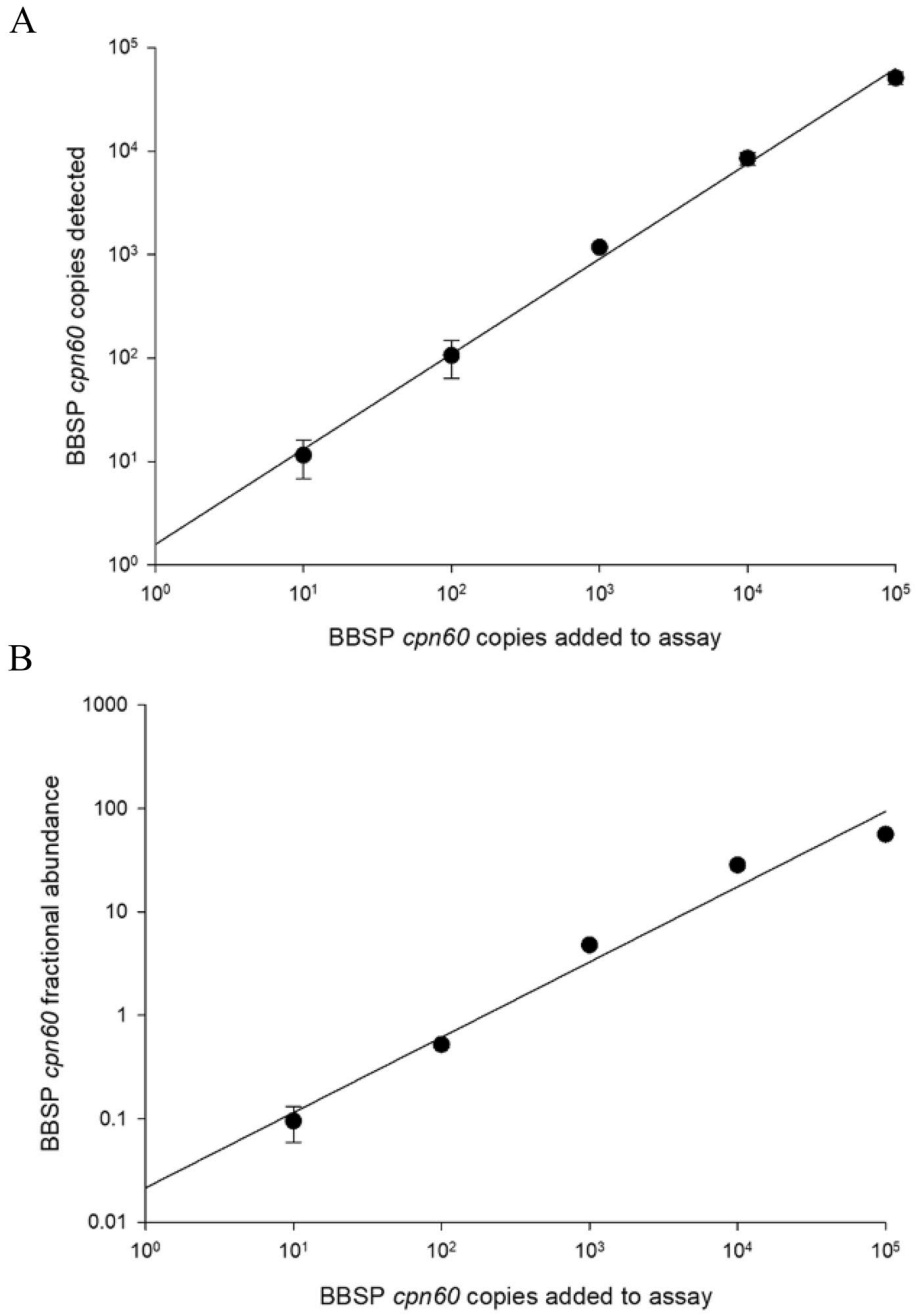
Quantification of BBSP *cpn60* in healthy blueberry tissues spiked with known copy numbers of BBSP *cpn60* plasmids. (**A**) Relationship between copies added and copies detected by ddPCR. (**B**) Fractional abundance (FA) of spiked BBSP *cpn60* in relation to blueberry DNA, as determined using the blueberry rubisco internal control assay.

The hydrolysis probe-based qPCR assay was adapted to the ddPCR format, including an internal standard based on the sequence of the *V. corymbosum* rubisco gene. To determine the linearity and accuracy of the ddPCR assay in this duplex format, a series of mock-infected standards were prepared consisting of DNA from uninfected blueberry leaves into which was added known, variable amounts of BBSP *cpn*60 plasmid DNA. Determination of the fractional abundance (FA) in each of these standards using ddPCR showed that the assay was highly linear over several orders of magnitude, although very high levels of BBSP were quantified somewhat less accurately (Fig. 1).

### LAMP assay targeting ‘*Candidatus* Phytoplasma asteris’ *cpn*60

To provide a detection assay that was suitable for screening large numbers of samples, a LAMP assay was developed targeting the *cpn*60 UT of BBSP. To determine its suitability for the detection of BBSP in field-collected samples, the following assay parameters were measured:

#### Analytical specificity

The ability of the LAMP and qPCR assays to detect various phytoplasmas was assessed using either plasmids containing cloned *cpn*60 UT PCR products, or total genomic DNA from infected plant tissues from a wide variety of phytoplasmas (Table 1). Positive results were observed only for samples corresponding to ‘*Candidatus* Phytoplasma asteris’, including subgroups 16SrI-E, -B, -R, -A, and -F (Table 1). All other phytoplasmas, corresponding to eleven other 16Sr groups and fourteen other ‘*Candidatus* Phytoplasma’ species, were negative with the LAMP assay. These results demonstrated the suitability of the LAMP assay for the specific detection of ‘*Candidatus* Phytoplasma asteris’-related strains, including BBSP (16SrI-E/AI)^27^. Similar results were observed for the qPCR assay, except that, in certain cases, the qPCR assay produced very weak signals with non-target phytoplasma species (Table 1).

**Table 1:** List of samples and/or *cpn60* UT clones used to measure the analytical specificity of the LAMP assay targeting BBSP. Plasmid DNA (10^7^ copies, containing the *cpn60* UT) amplified and cloned from plants infected with each of the specified groups and subgroups was used as template as described in the text. When available, total nucleic acids extracted from infected plant tissue was also tested, as indicated by (a). Complete descriptions of each of these strains can be found at http://cpnclassiphyr.ca.

Sample	*cpn60* UT group/subgroup (accession no.)	16Sr group/subgroup (accession no.)	Host	Phytoplasma species	LAMP		qPCR	
					T_p_, min:s (T_a_, °C)	Call	C_q_	Call
BBSP	*cpn60* UT I-IE (MH279496)	16SrI-E (MH279523)	*Vaccinium corymbosum* (blueberry)	*‘Ca.* P. asteris*’*	9:00 (82.44)	pos^a^	15.85	pos
SF1	*cpn60* UT I-IB (KJ940013)	16SrI-B (MH279534)	*Linum usitatissimum* (flax)	*‘Ca.* P. asteris*’*	8:00 (82.47)	pos^a^	16.86	pos
AY-col	*cpn60* UT I-IC (KJ939994)	16SrI-R (MH279535)	*Catharanthus roseus* (periwinkle)	*‘Ca.* P. asteris*’*	9:00 (82.77)	pos^a^	25.09	pos
O2L	*cpn60* UT I-IA (KJ939998)	16SrI-A (MH279536)	*Allium cepa* (onion)	*‘Ca.* P. asteris*’*	8:45 (82.47)	pos^a^	19.74	pos
CVB	*cpn60* UT I-IC (KJ939995)	16SrI-F (MH279545)	*Catharanthus roseus* (periwinkle)	*‘Ca.* P. asteris*’*	10:30 (82.43)	pos^a^	17.76	pos
S31b-GP-MA	*cpn60* UT XIII-IA (KT444666)	16SrXIII-A/D (KT444665)	*Fragaria* × *ananassa* (strawberry)	*‘Ca.* P. hispanicum*’*	ND^b^	neg^a^	ND^b^	neg
BN44948	*cpn60* UT XII-IA (KJ939979)	16SrXII-A (MH279537)	*Catharanthus roseus* (periwinkle)	*‘Ca.* P. solani*’*	ND^b^	neg^a^	ND^b^	neg
BGWL	*cpn60* UT XIV-IA (MH279492)	16SrXIV-A	*Cynodon dactylon* (Bermuda grass)	*‘Ca.* P.*’* cynodontis	ND^b^	neg	ND^b^	neg
PGY	*cpn60* UT V-IA (KJ939991)	16SrV-C (MH279539)	*Catharanthus roseus* (periwinkle)	‘*Ca*. P. ulmi’	ND^b^	neg	ND^b^	neg
AshY	*cpn60* UT VII-IA (KJ939978)	16SrVII-A	*Fraxinus* (Ash)	‘*Ca*. P. fraxini*’*	ND	neg	ND	neg
ChicBS	*cpn60* UT IX-IJ (KY986918)	16SrIX-J	*Cichorium intybus* (Chicory)	*‘Ca.* P. phoenicium*’*	ND^b^	neg^a^	ND^b^	neg
Cr	*cpn60* UT IX-IH (KJ939989)	16SrIX-A	*Catharanthus roseus* (periwinkle)	*‘Ca.* P. phoenicium*’*	ND^b^	neg	37.57	pos (weak)
JbWb190	*cpn60* UT II-IA (KY704478)	16SrII-D	*Simmondsia chinensis* (jojoba)	*‘Ca.* P. australasiae*’*	ND^b^	neg	ND^b^	neg
PeruPBT	*cpn60* UT XV-IB (MH279494)	16SrXV-B (KX810334)	*Carica papaya* (papaya)	*‘Ca.* P. brasiliense*’*	ND^b^	neg	38.01	pos (weak)
PYLR	*cpn60* UT X-IC (KJ940010)	16SrX-C (MH279541)	*Prunus persica* (pear)	‘*Ca*. P. pyri*’*	ND^b^	neg^a^	ND^b^	neg
AP	*cpn60* UT X-IA (KJ939977)	16SrX-A (MH279542)	*Malus domestica* (apple)	‘*Ca*. P. mali*’*	ND^b^	neg^a^	ND^b^	neg
ESFY	*cpn60* UT X-IF (KJ940007)	16SrX-F (MH279543)	*Catharanthus roseus* (periwinkle)	*‘Ca.* P.*’* prunorum*’*	ND^b^	neg^a^	ND^b^	neg
RS	*cpn60* UT V-IC(KJ939990)	16SrV-C (MH279544)	*Catharanthus roseus* (periwinkle)	‘*Ca*. P. rubi*’*	ND^b^	neg	38.22	pos (weak)
FD	*cpn60* UT V-IC (KJ939992)	16SrV	*Vitis vinifera* (grape)	‘*Ca*. P. ulmi*’*	ND^b^	neg	ND^b^	neg
SCWL	*cpn60* UT XI-ID (MH279493)	16SrXI-D (KC295286)	*Saccharum officinarum* (sugarcane)	*‘Ca.* P. oryzae*’*	ND^b^	neg	ND^b^	neg
R018	*cpn60* UT II-IC (MH279495)	16SrII-C (MH266698)	*Citrus* × *aurantiifolia* (lime)	*‘Ca.* P. aurantifolia’	ND^b^	neg	ND^b^	neg
TagTall	*cpn60* UT IV-IC (MH922781)	16SrIV-C (MH922778)	*Cocos nucifera* (coconut)	*‘Ca.* P. cocostanzaniae’	ND^b^	neg	ND^b^	neg
Sab4	*cpn60* UT IV-IE (MH922782)	16SrIV-D (MH922779)	Arecaceae (palm)	*‘Ca.* P. palmae’	ND^b^	neg	ND^b^	neg
Palm-ADR	*cpn60* UT IV-IE (MH922783)	16SrIV-E (MH922780)	*Cocos nucifera* (coconut)	*‘Ca.* P. palmae’	ND^b^	neg	ND^b^	neg

^a^These samples were also tested using DNA extracted from a plant infected with the same phytoplasma, which yielded the same result.

^b^*ND* not detected.

#### Analytical sensitivity

**Figure 2:**
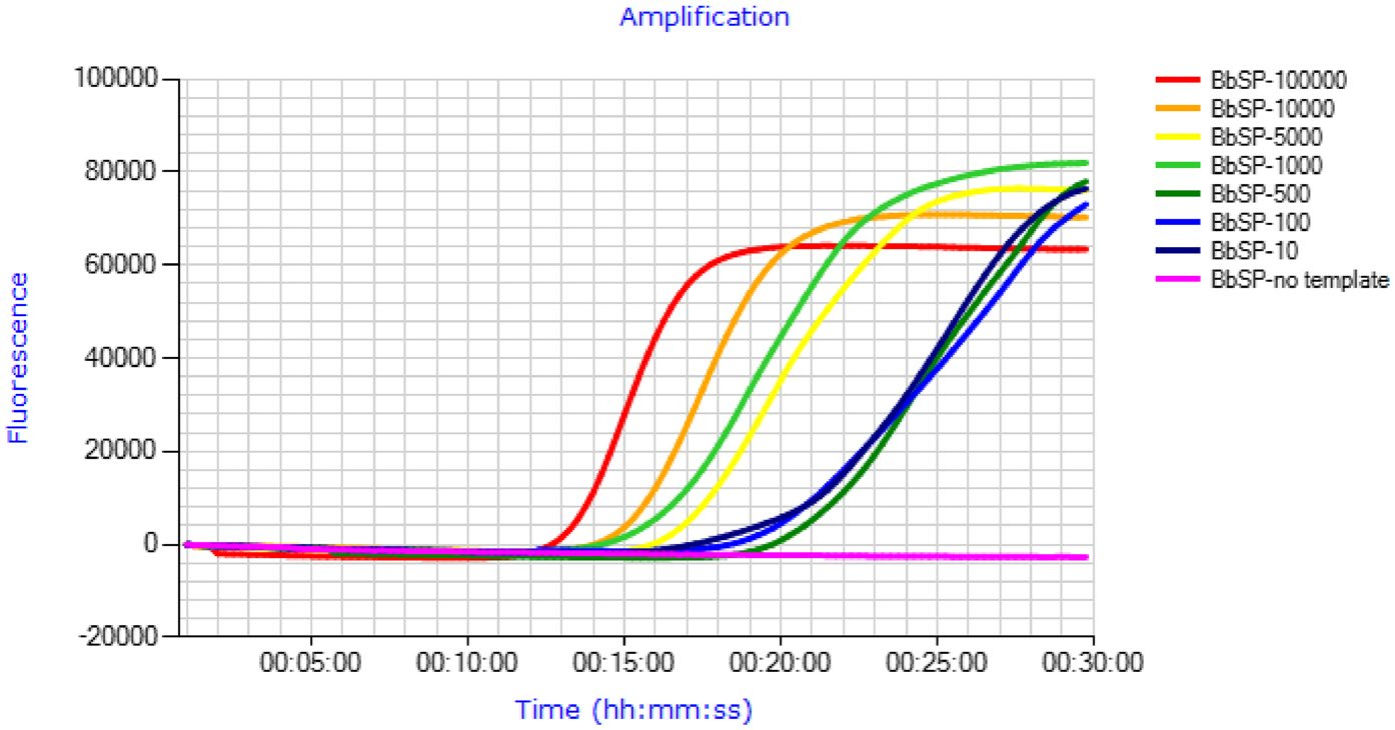
Detection of low levels of BBSP plasmid DNA in a background of uninfected blueberry DNA using LAMP. Templates consisted of the indicated copy numbers of BbSP *cpn60* UT plasmid.

The LAMP assay was capable of detecting low levels of BBSP DNA in a matrix of blueberry DNA in artificially inoculated samples (spiked), with as few as 10 copies of the BBSP *cpn*60 UT plasmid generating a positive result (Fig. 2). However, the linearity of the assay was low (Pearson r^2^ = 0.49). The limit of detection (LOD) of the LAMP assay, defined as the C_95_, or the concentration of analyte required to give a positive result in 95% of assays^29^, was determined in terms of FA using naturally infected blueberry tissue samples spanning the range of observed BBSP levels. Probit analysis indicated that the C_95_ of the LAMP assay corresponded to a FA of 0.463 in naturally infected samples. Similar results were obtained for the LAMP assay using an optimized curve-fitting model^30^, with a single-replicate 95% LOD of FA = 0.466 in naturally infected samples (Supplementary Table S3). This corresponded to a ddPCR-determined copy number of 60.2 (Supplementary Table S3). By contrast, the LOD for the qPCR assay, determined using plasmid templates, was 7.5 copies (Supplementary Table S3).

#### Precision

The repeatability of the LAMP assay was determined by examining the results of replicates of naturally infected samples at various ddPCR-determined levels of BBSP (Table 2). As expected, the assay results measured in time to positive (T_p_) were more reproducible (lower coefficient of variation) at higher levels of BBSP and less reproducible at lower levels. Similarly, the qPCR assay targeting BBSP demonstrated decreased repeatability at lower template levels (3.03% CV at 5 copies) compared to higher levels (1.10% CV at 10,000 copies—Supplementary Table S4). Overall, the repeatability of the C_q_ values of the qPCR assay was higher compared to the T_p_ values generated by the LAMP assay (Table 2; Supplementary Table S4).

**Table 2:** LAMP assay repeatability.

Sample	Tissue	Date	FA^a^	*cpn60* copies^b^	Mean T_p_, minutes	Standard deviation	n	CV, %
Qc4PR6-1	Leaf	2016-08-15	0.130	15	19.63	2.61	12	13.28%
Qc4PR6-1	Leaf	2016-07-12	0.423	45	20.14	2.30	12	11.42%
Qc1-P1L3	Leaf	2016-08-02	5.32	182	17.15	0.69	12	4.04%
NS1P4	Fruit	2016-09-13	30.13	7770	13.04	0.10	6	0.78%

^a^Fractional abundance of BBSP phytoplasma as determined by ddPCR; see text.

^b^*cpn60* copies/assay, measured by ddPCR; see text.

#### Linearity

**Figure 3:**
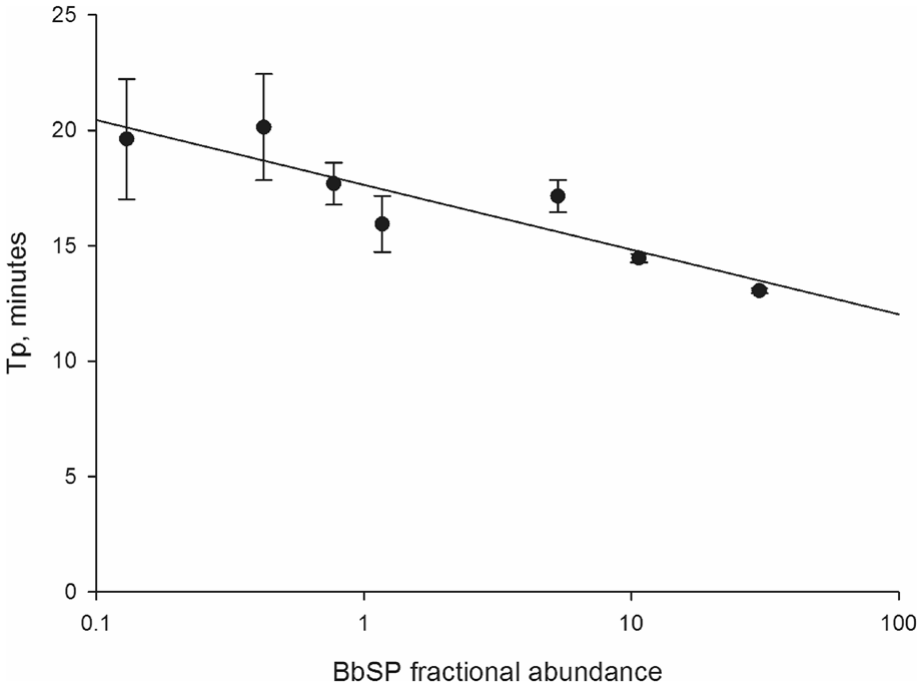
LAMP time to positive (T_p_) in relation to levels of BBSP in naturally infected blueberry samples. Each data point represents the mean of 6 or 12 measurements. Spearman correlation between ddPCR-determined FA and LAMP T_p_ was ρ = − 0.772, p < 0.0001, n = 40.

As expected, the LAMP assay displayed an inverse relationship between T_p_ and FA, with higher FA samples featuring a lower T_p_ than lower FA samples (Fig. 3). However, this relationship was not very strong (r^2^ = 0.8), which decreases the utility of the LAMP assay for quantifying BBSP in tissue samples. However, the Spearman correlation coefficient was highly significant (ρ = − 0.772; *p* < 0.0001), indicating a statistically significant relationship between BBSP amount and LAMP T_p_. Nevertheless, in light of these results the LAMP assay was ultimately implemented as a binomial, providing positive or negative results, rather than a quantitative assay. The qPCR assay (real-time format) displayed a very high correlation between BBSP *cpn60* copies and observed C_q_ (r^2^ = 0.9959—Supplementary Fig. S1), as is typically observed using qPCR. The same assay in ddPCR format also showed a high correlation between BBSP *cpn60* copies added to the assay and the calculated copy number (r^2^ = 0.998; Fig. 1).

#### Comparison of methods

The commonly accepted “gold standard” assay for phytoplasma detection is nested PCR targeting the F2nR2 fragment into the 16S rRNA-encoding gene^12,31–34^. To determine how the LAMP assay compared to this gold standard, we examined 256 blueberry tissue samples from Québec and Nova Scotia using both methods and compared the binomial results obtained by both assays for each sample. The large majority of samples that were positive by nested PCR were also positive using LAMP, providing a test sensitivity of 92% (Table 3). Conversely, the LAMP assay appeared to provide a relatively high rate of type I errors (false positives), with 30 blueberry tissue samples testing positive by LAMP but negative by nested PCR (Table 3), corresponding to a test specificity of 84.8%. However, when these 30 samples were examined with qPCR targeting BBSP *cpn*60 as well as a separate nested PCR assay targeting the ‘*Ca.* P. asteris’ *rp* gene^21^, all 30 samples were demonstrated to contain BBSP DNA (Supplementary Table S1). These results indicate that the higher rate of positives obtained using the LAMP assay reflected not type I errors (false positives) by the LAMP assay but type II errors (false negatives) by the nested PCR assay targeting the 16S rRNA-encoding gene. These nested PCR negative but LAMP positive samples tended to have a high C_q_ in the qPCR assay (mean C_q_ = 30), suggesting that this discrepancy may arise from a higher analytical sensitivity of the LAMP assay. Sequences were determined for samples that tested positive by 16S nested PCR but negative by LAMP, and all were shown to contain phytoplasma DNA (data not shown). Furthermore, the 168 samples examined that tested negative by both assays included all tissue types analyzed (leaves, fruits, stems, and roots), along with 28 that were taken from tissue (fruits or leaves) that was assessed as “healthy”. These results demonstrate that neither the LAMP assay nor the nested PCR assay generated spurious signals from uninfected blueberry tissues. We also examined the LAMP assay in comparison to another nested PCR targeting the *rp* operon of 16SrI (Aster Yellows) phytoplasmas^21^, which provided an assay sensitivity of 83% and specificity of 86% (Supplementary Table S5). The kappa statistic comparing the two assays (0.7) indicated a substantial level of agreement. For the qPCR assay, an assay sensitivity of 87% and specificity of 86% was determined compared to the Aster Yellows *rp* nested PCR assay (Supplementary Table S6), with a kappa statistic (0.7) that also indicated substantial agreement.

**Table 3:** Sensitivity and specificity of the LAMP assay, using nested PCR targeting the 16S rRNA-encoding gene as a gold standard. *95% CI* 95% confidence interval, calculated as described by Banoo et al.^25^.

Assay	16S nested PCR (F2nR2)			
LAMP	Positive	Negative	Total	
Positive	44	30	74	
Negative	4	168	172	
Total	48	198	246	

	Value	95% CI	Low	High
Test sensitivity	0.917	0.078	0.838	0.995
Test specificity	0.848	0.050	0.799	0.898

### BBSP surveys in Eastern Canada: 2016–2017

The validated LAMP assay was applied to symptomatic and asymptomatic plant tissues collected in 20 highbush blueberry fields in Nova Scotia and Québec in the 2016 and 2017 growing seasons. Four sites were sampled in Nova Scotia along with 16 sites in Québec (Supplementary File S1). At each site, multiple plants were sampled and tissues including leaves, fruits (when available), and in some cases woody stem and root were collected. Sites were visited at intermittent time points in each growing season for sample collection. In total, 197 tissue samples were collected from highbush blueberry plants in Nova Scotia in 2016 and another 209 samples in 2017. In Québec, where more fields were surveyed, 325 samples were taken in 2016 along with 987 in 2017. In total, 1718 samples were collected from 146 plants in Nova Scotia and Québec over the two growing seasons.

In Quebec, samples were taken on highbush blueberry plants belonging mainly to the cultivars Patriot, Blueray, Bluecrop and Duke or in lowbush blueberry plants (Supplementary File S1). In Nova Scotia, all samples were collected from highbush blueberry plants belonging to Liberty, Blue Gold, Duke and Burlington (Supplementary File S1). The results of the LAMP assays for BBSP on these samples are summarized in Tables 4 and 5, and the complete results are provided as a supplemental file (Supplementary File S2). In addition, the complete results of the BBSP surveys are available as an interactive map (R code and dataframe provided as supplemental file Distribution.zip).

**Table 4:** Summary of BBSP LAMP assay results on field samples taken in 2016.

Province	Site	Number of		Positive samples		Positive plants	
		Samples	Plants	Number	Proportion	Number	Proportion
Nova Scotia	NS1	80	5	29	36.25%	5	100.00%
Nova Scotia	NS2	85	5	6	7.06%	5	100.00%
Nova Scotia	NS6	19	4	15	78.95%	4	100.00%
Nova Scotia	NS7	13	3	3	23.08%	1	33.33%
Québec	Qc1	95	6	25	26.32%	4	66.67%
Québec	Qc2	82	5	0	0.00%	0	0.00%
Québec	Qc3	76	5	0	0.00%	0	0.00%
Québec	Qc4	72	10	14	19.44%	2	20.00%
	Total	522	45	92		21

**Table 5:** Summary of BBSP LAMP assay results on field samples collected in 2017.

Province	Site	Number of		Positive samples		Positive plants	
		Samples	Plants	Number	Proportion	Number	Proportion
Nova Scotia	NS1	104	5	14	13.46%	1	20.00%
Nova Scotia	NS2	105	5	0	0.00%	0	0.00%
Québec	Qc1	58	6	15	25.86%	4	66.67%
Québec	Qc2	55	5	1	1.82%	0	0.00%
Québec	Qc3	39	5	0	0.00%	0	0.00%
Québec	Qc4	84	9	24	28.57%	4	44.44%
Québec	Qc6	33	3	0	0.00%	0	0.00%
Québec	Qc7	62	5	0	0.00%	0	0.00%
Québec	Qc8	96	7	36	37.50%	4	57.14%
Québec	Qc9	57	10	15	26.32%	3	30.00%
Québec	Qc10	65	6	0	0.00%	0	0.00%
Québec	Qc11	50	4	0	0.00%	0	0.00%
Québec	Qc12	58	6	0	0.00%	0	0.00%
Québec	Qc13	74	5	30	40.54%	4	80.00%
Québec	Qc14	72	5	13	18.06%	1	20.00%
Québec	Qc15	66	5	7	10.61%	1	20.00%
Québec	Qc16	66	5	0	0.00%	0	0.00%
Québec	Qc17	52	5	0	0.00%	0	0.00%
	Total	1196	101	155		22	

BBSP was found in most of the fields surveyed in both provinces in 2016, with only 2 of 8 sites determined to lack the pathogen in all samples collected (Table 4). In some fields, the majority (up to 100%) of the plants were positive in at least one sample taken, but not all samples taken from BBSP positive plants were positive at all times (Supplementary File S2). In total, 21 plants were identified in all fields examined in 2016 that were positive for BBSP.

In 2017, more sites in Québec and fewer sites in Nova Scotia were surveyed. The expanded survey area in Québec identified 5 more sites that contained BBSP positive plants, and another 7 sites where the pathogen was not detected (Table 5). At site Qc2, all samples had tested negative for BBSP in 2016, but a single sample taken in 2017 from plant Qc2P2 was faintly positive in the fruit at one time point (2017-08-30). All other samples taken from that plant were negative in all tissues in 2016 and 2017 (Supplementary File S2). The 2017 field surveys identified 22 BBSP positive plants at one site in Nova Scotia and 7 sites in Québec.

**Figure 4:**
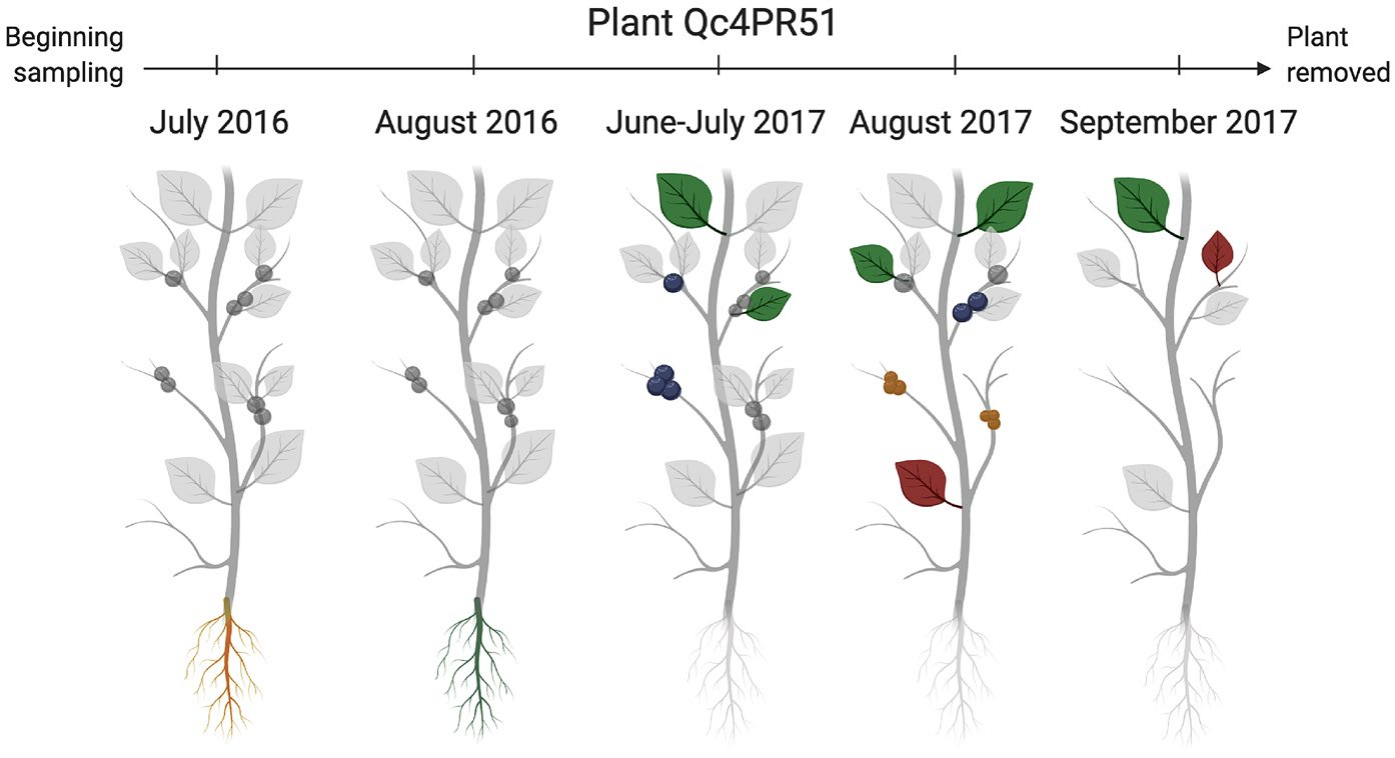
Outcome of tests on samples taken on a single highbush blueberry plant (Qc4PR5-1, cultivar Blueray,) in 2016 and 2017. Legend: red root (+); green root (−); red fruit (+); blue fruit (−); red leaf (+); green leaf (−). Gray tissues (roots, fruit, leaves) were not tested.

At least one sample representing each of the tissue types examined was determined to be positive in at least one of the plants. Many of the BBSP positive plants had the pathogen in the leaves and fruits, and of the four plants from which woody stem tissue was sampled, only plant Qc1P1L3 was positive in this tissue (at all time points analyzed). This plant was particularly symptomatic and produced fruit only at the earliest time point analyzed (2016-06-28) with only a limited number of leaves produced thereafter (Supplementary File S2); ultimately this plant was removed by the producer. Three plants had root tissues sampled and examined for BBSP. One of these plants, Qc4PR51, was positive in the root at a single time point (2016-07-19) but negative at two time points later in the 2016 growing season (Fig. 4). While no other tissues were sampled from this plant in 2016, Qc4PR51 displayed evidence of BBSP infection in the 2017 growing season in the leaves and fruit in later season time points, starting 2017-08-16 in the fruit (Fig. 4).

### 2018 field surveys

**Figure 5:**
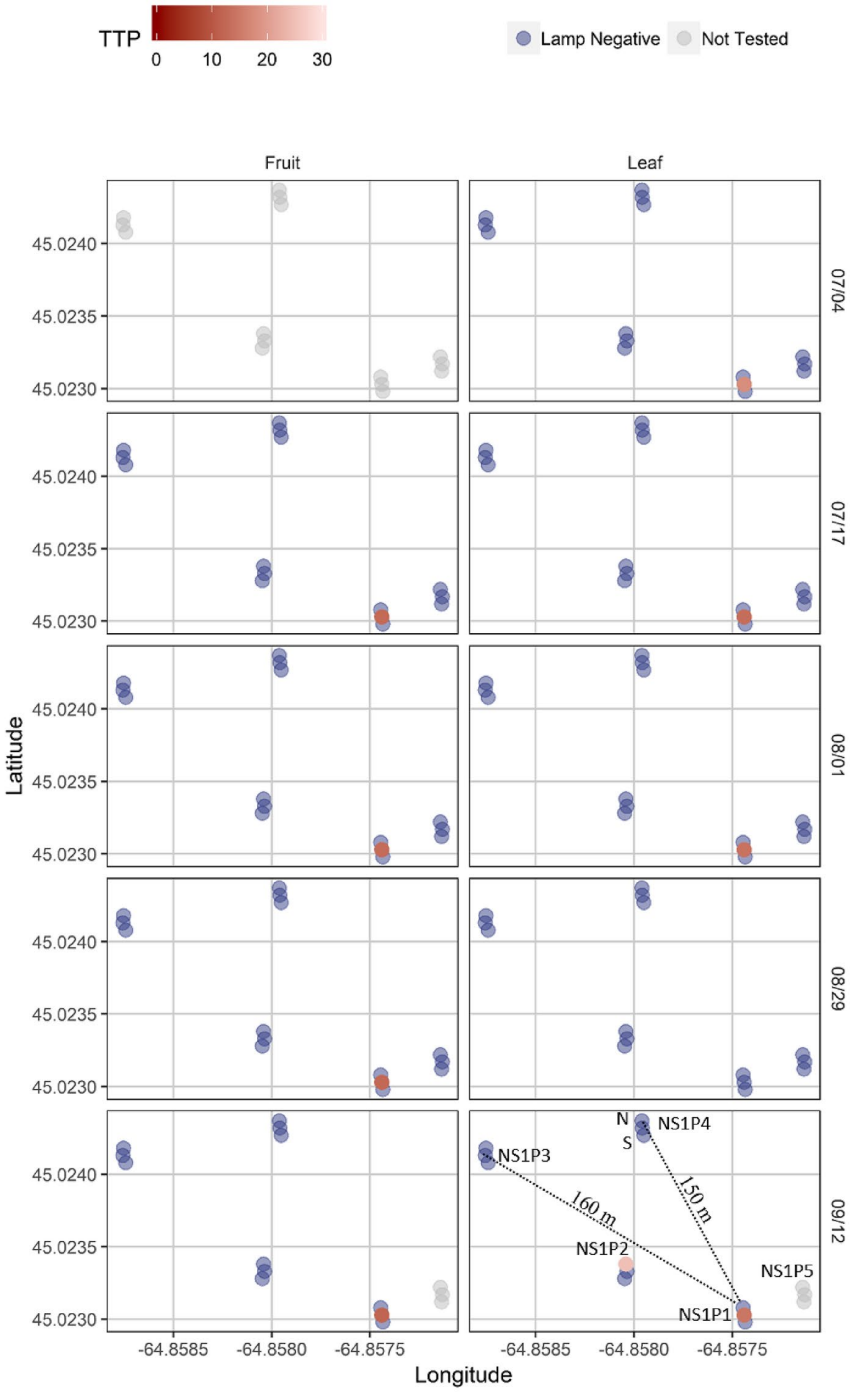
LAMP results on plants located at site NS1 close to a chronically infected plant (NS1P1). Sampling dates (month/day) are from 2018.

In the final sampling year, the samples were collected to address the question of whether or not BBSP was observed to spread from infected plants to nearby plants within fields. At Nova Scotia site NS1, 5 plants separated by up to 150 m were examined, along with plants immediately adjacent to them. One of the plants, NS1P1, had been identified as BBSP positive in leaves and fruit in previous sampling years (2016 and 2017). Plants in the vicinity of NS1P1 were sampled across the growing season and examined for BBSP. Despite years of close proximity to a highly infected plant (NS1P1, which was positive at all time points analyzed), none of the 14 plants located within approximately 150 m of the infected plant displayed any evidence of BBSP infection in leaves or fruits across the growing season in 2018 (Fig. 5).

**Figure 6:**
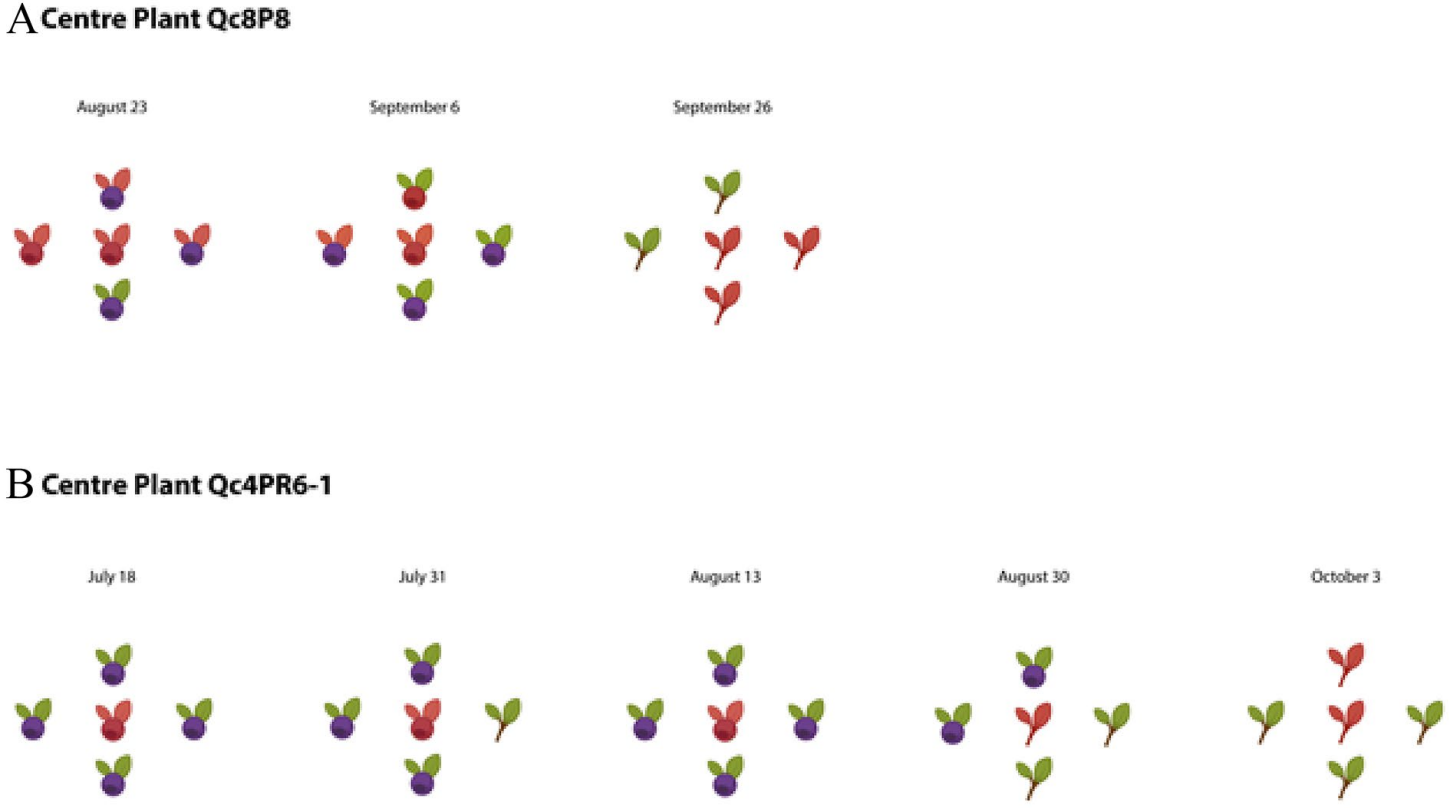
Outcome of LAMP tests on samples taken on a highbush blueberry plant and four nearby highbush blueberry plants in 2018. The plants were 1.5 and 2 m distant respectively on the row (vertically) and between rows (horizontally). (**A**) Centre plant = Qc8-P8. All plants were of cultivar Bluecrop. (**B**) Centre plant Qc4-PR6-1 was known to be phytoplasma positive since 2016. All plants were of cultivar Blueray. Legend: red root (+); green root (−); red fruit (+); blue fruit (−); red leaf (+); green leaf (−). Gray tissues (roots, fruit, leaves) were not tested. See Plate 1 for selected plants.

In Québec, two known BBSP-infected plants and four neighboring plants each were sampled throughout the growing season at two separate sites (Supplementary Fig. S2). At one site, the infected plant (Qc8P8) remained BBSP positive in both leaves and fruit throughout the growing season, while the four neighboring plants sampled demonstrated intermittent BBSP presence (Fig. 6). At the other site, the BBSP-infected plant (Qc4 PR6-1) also remained positive over several months, while three of the neighboring plants remained BBSP-free. Late in the season, one neighboring plant tested positive for BBSP (Fig. 6).

### Determination of pathogen load in infected plants

**Figure 7:**
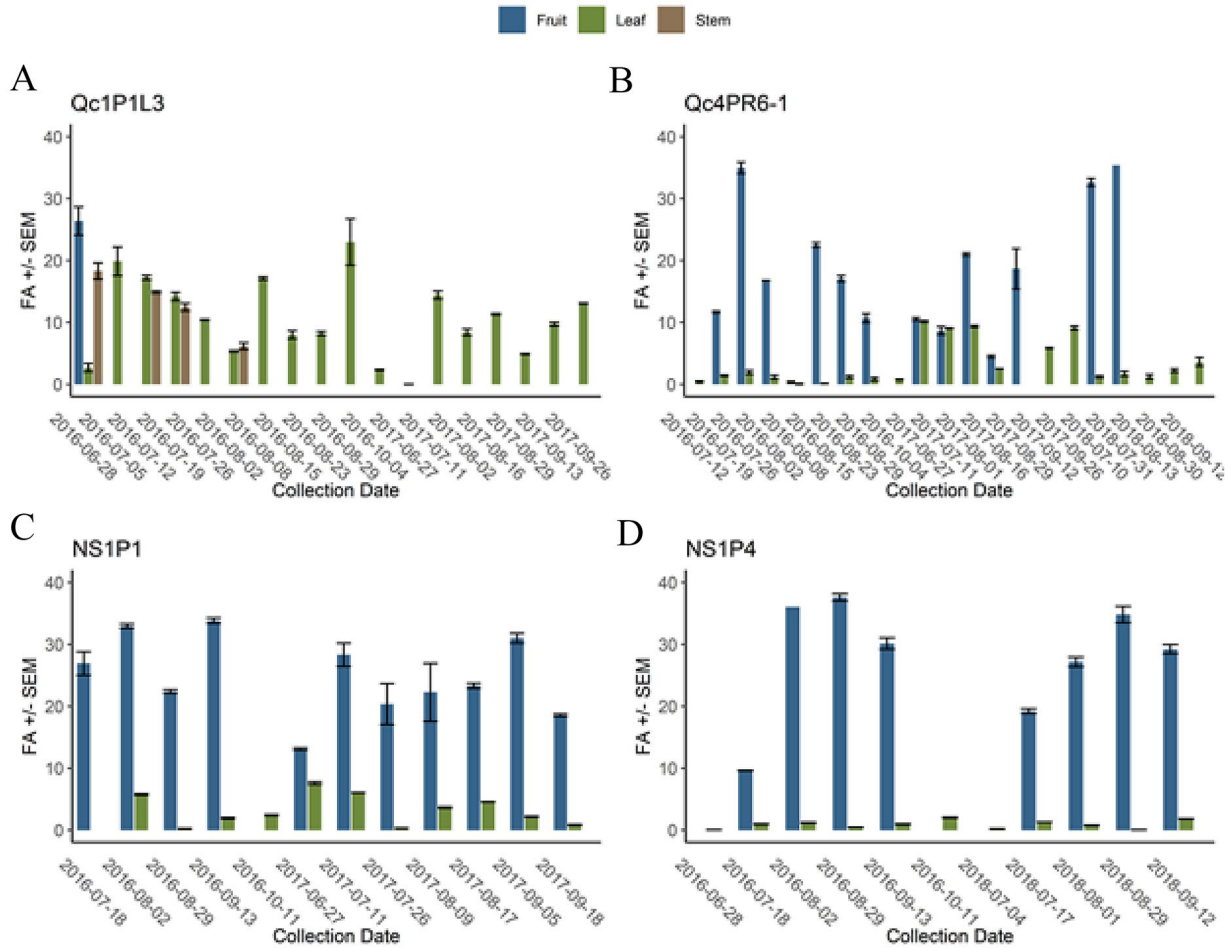
ddPCR results on highbush blueberry plants (**A**) Qc1P1L3 cultivar Blueray 2016 and 2017, (**B**) Qc4PR6-1 cultivar Blueray 2016, 2017 2018, (**C**) NS1P1 cultivar Liberty 2016 and 2017 and (**D**) NS1P4 cultivar Blue Gold 2016 and 2018. Fractional abundance in fruit (blue), leaves (green) and stem (brown).

BBSP was quantified by ddPCR in a series of tissue samples taken from symptomatic blueberry plants in Québec and Nova Scotia over two or three growing seasons. Two plants were examined in Québec, which were at differing stages of blueberry stunt disease. One of these plants, Qc1P1L3, was highly symptomatic and provided only a single time point for BBSP quantification in fruit, because at all subsequent time points no fruit was produced. The FA of BBSP in the single fruit sample that was available was the highest observed in any of the leaf or stem samples, but not dramatically higher (Fig. 7A). BBSP levels in the leaf and stem samples taken subsequently remained rather high throughout 2016 and 2017 (leaf only) until the plant was removed and destroyed by the producer (Fig. 7A). In contrast, plant Qc4PR6-1 had BBSP levels that were consistently much higher in fruits compared to leaves in 2016, but the levels were closer to those in fruits and leaves in 2017, mainly due to an increase in BBSP in leaf tissue (Fig. 7B). In 2018, the levels of BBSP decreased in leaf tissues but remained high in the three fruit samples analyzed from early July to mid-August (Fig. 7B). Similarly, the two plants analyzed from Nova Scotia also displayed far higher levels of BBSP in fruits compared to leaves throughout the sampling seasons 2016–2018 (Fig. 7C,D).

The BBSP levels in the leaves were not only typically much lower than those found in the fruits but also tended to fluctuate across the sampling seasons, and sometimes strongly. For example, BBSP in the leaves of plant Qc4PR6-1 was relatively low in 2016, much higher in 2017, and low again in 2018 (Fig. 7B). These fluctuations were also observed within a sampling season in the leaf tissue—plant NS1P4 had a BBSP FA of ~ 0.2–1.3 in early July to mid-August of 2018, but the sample taken on August 29 had an approximately tenfold decrease in BBSP (FA ~ 0.01), then increased again to the levels seen earlier in the summer in the September 12 sample (Fig. 7D).

## Discussion

The concept of biovigilance for crop disease management encompasses several interlinked aspects along a continuum of research foci, which begin with disease awareness and detection/identification^11^. In the case of BBSP, it is important for producers to become aware of the presence of the disease in the area, since the symptoms are variable and unreliable to make a decision, and the disease is spread by phytophagous insects. It is therefore important to have in place tools that facilitate the detection of the disease in infected plant and insect tissue. To be effective, disease detection should be as rapid and accurate as possible, and users should have confidence that detection assays have been thoroughly validated and produce results that are reliable and easily interpreted. In order to meet this need, we have designed and validated a LAMP-based diagnostic for BBSP and applied this assay to field-collected blueberry samples from two Canadian provinces over three years. The validation was carried out according to performance specifications set out in Burd’s validation of laboratory-developed molecular assays for infectious diseases^29^, including the establishment of analytical specificity, analytical sensitivity, precision, linearity and accuracy. The LAMP diagnostic specifically detected the 16SrI ‘*Candidatus* Phytoplasma asteris’ group that includes BBSP, and these results indicate the potential utility of the assay to detect other subgroups within the geographically widely distributed 16SrI (Aster Yellows) group. The precision of this assay was demonstrated by the repeatability of multiple replicates of naturally infected samples at various levels of BBSP. The LAMP assay exhibited an inverse, albeit weak, relationship between time to positive and fractional abundance. To establish accuracy compared to established methods, a comparison of methods was carried out, comparing the LAMP diagnostic with nested PCR targeting the 16S rRNA gene, a traditional gold standard diagnostic for this disease. In addition to its vastly increased speed and ability to target 16SrI phytoplasmas, the LAMP assay was found to have higher sensitivity compared to the 16S nested PCR, as samples that had tested positive by LAMP but negative by 16S nested PCR were demonstrated to be true positives using PCR assays targeting other genes.

The performance specifications determined by the BBSP LAMP validation compare well with other recently described LAMP assays. The improved sensitivity of LAMP over PCR is typical, as LAMP is less prone to inhibition than PCR. This has also been recently shown with regards to the development of a bacterial meningitis LAMP assay that was found to be between 10 and 10,000 times more sensitive than PCR^35^ and another LAMP assay targeting *Puccinia triticina* causing leaf rust of wheat 500-fold greater sensitivity than PCR^36^. The analytical specificity of LAMP targeting the potato pathogen *Dickeya dianthicola* also showed high specificity, with 16 different *D. dianthicola* strains being amplified while 56 other bacterial strains were not^37^. The time to positive found by Castro et al.^38^ investigating a rapid diagnosis of Zika virus was found to be in the same 13–15 min range as our work described here. An inverse relationship between time to positive and input copy number that was linear over several orders of magnitude was also demonstrated for a Strawberry Green Petal phytoplasma (16SrXIII) LAMP assay^15^.

The fact that LAMP does not require thermal cycling makes it a simple, fast and cost-effective method compared with PCR. Moreover, because LAMP is isothermal, it is possible to deploy the assay to remote, potentially resource-limited settings for nearly immediate, on-site diagnostics. Such an approach would greatly facilitate the rapid and accurate detection of BBSP in infected plant tissue. While plant tissue presented a relatively abundant source of infected material for the development and validation of this assay described here, a true biovigilance-based approach, which seeks to detect and mitigate BBSP infections before they become a problem, may require the examination of known leafhopper vectors collected in or near blueberry fields. This would facilitate the detection of potential carriers before they transmit disease. On-site detection of BBSP would require a suitable means of extracting DNA from plant or insect tissue. To facilitate this, FTA matrix cards, which are designed for DNA extraction from plant tissue, have been adapted to detect phytoplasma infections in insect tissue^39^. The suitability of LAMP coupled with rapid and inexpensive nucleic acid purification and colorimetric detection for rapid and accurate detection of SARS-CoV2 has also been recently described^40^, demonstrating the utility of this approach for pathogen detection in the context of human disease.

Despite the many advantages of this technique, there are limitations to the use of LAMP as a diagnostic tool. The LAMP amplicon is stable, and the method features a high analytical sensitivity, and so can easily cross-contaminate, leading to false positive results. Additionally, primer design can be challenging as the use of multiple primers can necessitate manual primer design and can also increase the chance of primer–primer hybridizations. Hence, it is often necessary to examine multiple LAMP primers to find a set that produces suitable results, and thorough validation of assay performance is essential. While direct sequencing of LAMP products for disease detection has been developed in the context of covid (LamPORE)^41^, the technique does not generally lend itself to this approach for amplicon detection, nor does the timing of the positive signal reliably indicate quantity. Therefore, other tests must be undertaken to confirm sequence identity or quantify pathogen load. Recently described, alternative isothermal amplification methods such as Recombinase Polymerase Amplification have been shown to generate results with comparable speed and sensitivity, but simpler primer design than LAMP^42^.

Having developed and validated a suitable rapid, low-cost assay, we applied it to determine the geographic distribution of BBSP in the Eastern Canadian provinces Québec and Nova Scotia. Surveys of nearly 2000 samples indicate that BBSP was widespread throughout the sampling regions in both provinces throughout the course of multiple growing seasons. Only the most severely stunted and unproductive plants warranted destruction by the producer.

To quantify BBSP levels in infected plants across time, we developed and applied a ddPCR assay. Application of this assay to select survey samples showed that BBSP fractional abundance changes over time. In some blueberry plants, BBSP levels in leaves were observed to drop below the detection limit only to re-appear at later time points in the season. Other phytoplasmas have been shown to be continually in flux within plants and over time, such as Chrysanthemum yellows phytoplasma in *Chrysanthemum carinatum*^43^ and ‘*Candidatus* Phytoplasma aurantifolia’ in lime^44^. In this work, blueberry leaf tissue was consistently found to have lower BBSP fractional abundance compared to fruit tissue. This is consistent with some phytoplasmas preferentially being found in reproductive tissues^45^. Although higher levels of BBSP are found in fruit, leaves are more continuously available, and a more convenient substrate for DNA extraction; therefore, we recommend that in Eastern Canada early August would be the optimal time for sampling blueberry leaves for BBSP. This time of the growing season is also suitable for screening blueberry plants for viruses known to co-infect with BBSP, such as Blueberry Red Ringspot Virus and Tobacco Ringspot Virus^8^.

Only the most severely symptomatic blueberry plants were found to have very high fractional abundance of BBSP. Although very low levels of BBSP may not be associated with economic losses, and may therefore not yet warrant management interventions, knowledge about the presence of BBSP remains useful for tracking distribution, spread, disease development or evaluating efficacy of IPM programs, especially where insect vectors are present. To address whether or not BBSP is spread between neighboring plants, we examined four plants immediately adjacent to a single plant known to have a high fractional abundance of BBSP. Some evidence of possible spread between neighboring plants was detected in one site in Québec (Fig. 6), although further investigation will need to be carried out to answer this question definitively. In the field examined in Nova Scotia, no evidence of spread to neighboring plants within a radius of approximately 150 m was observed. These results suggest that the spread of the phytoplasma infection to neighboring plants was neither rapid nor widespread, which may be the result of insect control measures undertaken by producers. It is possible that the phytoplasma we detected in the production fields was present in the plant material upon importation and was undetected when the plants were first established. However, the origin of the disease in eastern Canada is still unknown.

We have designed and validated the first *cpn60*-targeted LAMP assay for aster yellows phytoplasma and applied it to BBSP detection in blueberry tissues, which provides the fastest and least costly in-field option for detection that has been described to date. All molecular diagnostic tests have strengths and weaknesses, and assay choice will necessarily involve trade-offs. The high analytical specificity of the LAMP method will provide answers to very specific diagnostic questions (presence of 16SrI phytoplasmas), and therefore its use as a broad screening tool for diseases of unknown etiology is limited. In situations where assay mobility is required, but cost is less important, sequence-based methods such as Nanopore can provide more open-ended diagnostic information in cases where disease etiology is unknown or mixed infections may be suspected^46^. Moreover, Nanopore can provide sequence information outside of the targeted region, thereby providing additional taxonomic and genotypic data that can inform disease management decisions. Future work will deploy this technology to Eastern Canada to diagnose co-infection of blueberries and other crops with phytoplasmas and various viral or fungal pathogens as part of a biovigilance-based approach to disease management.

## Materials and methods

### Study areas and sample collection

In Nova Scotia, commercial highbush blueberry fields, located within the Annapolis Valley, were selected based on symptoms. Sampling was conducted monthly throughout the growing seasons. Locations of individual plants were recorded using a hand-held GPS unit and marked with flagging tape. Plants were imaged to capture symptoms. At each location, 5 plants were 1 young leaf near the bottom, 1 young leaf near the top, 1 old leaf near the bottom and one old leaf near the top. Additional samples included flowers, berries and stems. Plant and insect samples were placed in Styrofoam boxes with freezer packs and shipped to AAFC Saskatoon, Saskatchewan on the day of sampling or within 24 h of sampling. In Québec, plants were collected from 23 localities, and were selected and sampled in similar fashion to those in Nova Scotia (Supplementary File S1).

Sample collection complied with Canadian federal guidelines and legislation. Moreover, field access was granted and permission to collect samples was obtained from participating blueberry growers in all the fields studied.

### DNA extraction

Blueberry leaves, stems, roots and fruits were collected, lyophilized for 48 h, and DNA was extracted using the Qiagen DNeasy 96 Plant kit (Hilden, Germany) according to manufacturer’s instructions. At least two wells per plate were processed without a sample, serving as extraction blanks to monitor possible cross-contamination.

### qPCR assay development

The known sequence of the BBSP *cpn60* UT (GenBank KU523402)^21^ was used to design PCR primers and a hydrolysis probe for BBSP detection and quantification with Beacon Designer v7 (Premier Biosoft, Palo Alto, CA). The sequences of the amplification primers and hydrolysis probe are provided in Supplementary Table S2. All primers and hydrolysis probes were synthesized by Integrated DNA Technologies (Coralville, IA). Thermal cycling conditions for real-time qPCR were optimized using gradient PCR on a C1000 thermal cycler base with a CFX96 qPCR module (Bio-Rad) (Supplementary Table S2). The qPCR assay used 300 nM of each primer, 200 nM of hydrolysis probe, and 1 × SSoAdvanced Universal Probes Supermix (Bio-Rad). The efficiency of the qPCR assay was determined using a dilution series of the BBSP *cpn60* plasmid corresponding to known copy numbers and determining the C_q_ of each template amount, then calculating PCR efficiency (E) as described by Pfaffl^34^.

The hydrolysis probe-based assay for BBSP was adapted to the droplet digital PCR format using primer (900 nM) and probe (250 nM) concentrations suggested in the BioRad ddPCR applications guide (2014 version). In addition, to facilitate the determination of results in terms of fractional abundances (FA), a second hydrolysis probe assay was developed based on the *V. corymbosum Rubisco* gene (GenBank KJ773964.1) (Supplementary Table S2). Amplifications used 1 × ddPCR Supermix for Probes (Bio-Rad) and a template volume of 2 µl in a total volume of 20 µl. Template DNA was digested prior to assay using *Eco*RI (genomic DNA samples) or *Pst*I (BBSP *cpn60* plasmid samples) at 37 °C for 60 min. Amplification conditions for ddPCR were optimized using gradient PCR on a BioRad C1000 Touch thermocycler (Supplementary Table S2). Droplets were generated using a QX100 droplet generator (Bio-Rad), and amplifications used a C1000 Touch thermocycler (Bio-Rad), Droplets were analyzed post-amplification using a QX100 droplet reader (Bio-Rad), and results were calculated using QuantaSoft v.1.6.6 (Bio-Rad).

### LAMP assay

To provide a rapid and inexpensive assay for screening large numbers of samples, an aster yellows (AY) phytoplasma *cpn60* sequence (GenBank KJ940013) was used to design primers with LAMP Designer software v.1.13 (Premier Biosoft). The sequences of the amplification primers are shown in Supplementary Table S2. Primers were synthesized by Integrated DNA Technologies and the FIP (forward internal) and BIP (backward internal) primers were purified by HPLC. LAMP assays used 200 nM each of SF1-F3 and SF1-B3, 1600 nM each of SF1-FIP and SF1-BIP, and 800 nM each of SF1-loopF and SF1-loopB. Amplifications were performed in a final volume of 25 µl with 2 µl of template DNA and used 1 × WarmStart LAMP colorimetric or fluorometric mastermix (New England Biolabs, Whitby, ON). Alternatively, LAMP reactions were performed using 1 × Isothermal Mastermix (Prolab Diagnostics, Richmond Hill, ON). LAMP products were generated using a C1000 Touch thermocycler (Bio-Rad) and detected visually in the case of the colorimetric mastermix or using a Genie II/III instrument (Prolab Diagnostics) or CFX96 qPCR module on a C1000 thermocycler base (Bio-Rad) for fluorometric reactions. LAMP reactions proceeded for 40 min (fluorescent detection) or 60 min (colorimetric detection) at 63 °C and fluorescent reactions were followed by an annealing protocol to determine the annealing temperature (T_a_) of the amplified product (Supplementary Table S2).

### Validation of the LAMP assay

The performance parameters of the LAMP assay that was developed for the detection of BBSP in plant tissues were evaluated according to recommended standards for evaluating molecular diagnostic assays^24^. Specifically, the following parameters were assessed:

#### Analytical specificity

The ability of the LAMP assay to discern AY phytoplasmas, including BBSP, from other phytoplasmas was assessed using as template genomic DNA isolated from infected plants, and/or plasmid DNA corresponding to the cloned *cpn60* amplified from characterized phytoplasma strains.

#### Analytical sensitivity

To examine the ability of the AY LAMP assay to detect low levels of BBSP DNA, a series of mock-infected samples was prepared in which BBSP plasmid DNA was spiked into a matrix of uninfected blueberry DNA. LAMP assays were performed on each sample in both the fluorimetric and colorimetric formats. The LAMP assay limit of detection (LOD) was assessed using a series of naturally infected blueberry plant tissues in which the fractional abundances of BBSP had been measured using ddPCR. Selected samples that spanned the full range of FA observed in 41 naturally infected samples (FA = 0.13–30.1) were assayed by LAMP 6–12 times each (total of 72 replicate assays) using fluorescent detection on a Genie instrument, and the proportion of positive results at each FA was determined. The LOD was specified as the C_95_, or the concentration of BBSP that resulted in a positive result in 95% of assays^24^, and was calculated using probit analysis (SPSS v.24). LOD was also measured using a series of curve-fitting models that report the LOD as the concentration of analyte resulting in a 95% single-replicate detection rate using the best-fitting model^30^. Results were determined on naturally infected samples for LAMP (reported as FA and as the ddPCR-determined *cpn60* copy number in the sample) and on plasmid templates for qPCR.

#### Repeatability

Assay precision was determined by examining the time to positive (T_p_) generated from all of the replicates of the naturally infected samples used for LOD determination. Repeatability was determined by calculating the coefficient of variation of the T_p_ for each of four levels of BBSP. The repeatability of the qPCR assay was determined by measuring the C_q_ values obtained at various levels of *cpn60* plasmid template DNA (5 copies, 100 copies, 1000 copies) at 12 replicates each, then determining the coefficient of variation as for the LAMP assay.

#### Linearity

The relationship between the BBSP FA and the T_p_ was examined by polynomial regression analysis of the T_p_ determined at each level of BBSP in naturally infected samples. For qPCR, the linearity was determined in the same manner, but using plasmid templates.

#### Comparison-of-methods

The clinical sensitivity and specificity^25^ of the LAMP assay was evaluated by comparison to an acknowledged gold standard for phytoplasma detection, nested PCR targeting the 16S rRNA-encoding gene^7^. To amplify this target from phytoplasmas, primers P1^35^ and P7^36^ were used in an initial round of PCR to generate a product of ~ 1.8 kb. The product of this PCR was diluted 1:30 and 2 µl used as a template in a second round of PCR with primers R16F2n and R16R2^7,9^. Both rounds of PCR used 2.5 mM MgCl_2_, 500 nM of each dNTP, 400 nM of each primer and 1 U *Taq* DNA polymerase (Invitrogen). Thermal cycling conditions for both rounds were 95 °C, 10 min (1×); 95 °C, 1:00/55 °C, 1:00/72 °C, 1:45 (35×); 72 °C, 10:00 (1×). Samples were analyzed on a 1.5% agarose gel and were characterized as positive or negative based on the appearance of a PCR product of the expected size (1.2 kb). The same samples were analyzed using the BBSP-targeted LAMP assay as described below. Results were analyzed using a 2 × 2 table and sensitivity and specificity along with 95% confidence intervals calculated as described^25^. Samples that were discordant (LAMP positive/nested PCR negative) were re-analyzed using BBSP-specific real-time qPCR as described here (Supplementary Table S2) as well as using nested PCR targeting the ribosomal protein (*rp*) operon^15^. For *rp* operon amplification, primers rpF1/rpR1^37^ were used in the first round, then the PCR product was diluted 1:30 and 2 µl used in a second round with primers rp(I)FIA/rp(I)RIA^38^. PCR and thermal cycling conditions for *rp* (both rounds) were as reported by Lee et al.^38^, except 1U Platinum *Taq* polymerase (Invitrogen) was used per reaction. In some cases the second-round *rp* PCR products were directly sequenced using the amplification primers (Eurofins Genomics, Toronto, ON). LAMP and qPCR assay results were compared to the AY-targeted *rp* assay by setting the *rp* assay results as the gold standard and calculating the sensitivity and specificity in the same manner.

### LAMP assays on field-collected samples

LAMP reactions were performed on DNA extracted from field-collected blueberry plant tissue using 2 µl of template in a 25 µl reaction volume. Initial screens used colorimetric detection and samples that tested negative (remained pink) were not examined further. Samples that yielded a positive result (yellow) or an intermediate result (orange) were subsequently examined by fluorimetric LAMP. Samples were considered positive if they provided a positive result in the secondary screen, with a T_a_ of the amplified product within 0.8 °C of that of the positive control (product generated from BBSP *cpn60* plasmid DNA added at 10^5^–10^6^ copies per reaction). All assays included “no template” controls with water in place of template DNA.

## Supplementary Information

Supplementary Information 1.

Supplementary Information 2.

Supplementary Information 3.

Supplementary Information 4.

Supplementary Information 5.
