# Supplementary material for: Detection of blueberry stunt phytoplasma in Eastern Canada using cpn60-based molecular diagnostic assays
Source: Sci Rep. 2021 Nov 11;11:22118. doi: 10.1038/s41598-021-01439-4 (PMC8586236; doi:10.1038/s41598-021-01439-4)
Supplement: Supplementary file 5 — Supplementary Information 5. [file 41598_2021_1439_MOESM5_ESM.docx]

Detection of blueberry stunt phytoplasma in Eastern Canada using *cpn60*-based molecular diagnostic assays

Christine Hammond^1^, Edel Perez-Lopez^2^, Jennifer Town^1^, Charles Vincent^3^, Debra Moreau^4^, Tim Dumonceaux^1*^

^1^Agriculture and Agri-Food Canada, Saskatoon, Saskatchewan, Canada

^2^Université Laval, Faculté des Sciences de l’Agriculture et de l’Alimentation, Département de Phytologie, Québec City, Québec Canada

^3^Agriculture and Agri-Food Canada, Saint-Jean-sur-Richelieu, Québec, Canada

^4^Agriculture and Agri-Food Canada, Kentville, Nova Scotia, Canada

*Corresponding author

E-mail: tim.dumonceaux@agr.gc.ca (TD)

SUPPLEMENTAL INFORMATION

Figure S1. Linearity of the BBSP qPCR assay in real-time format.


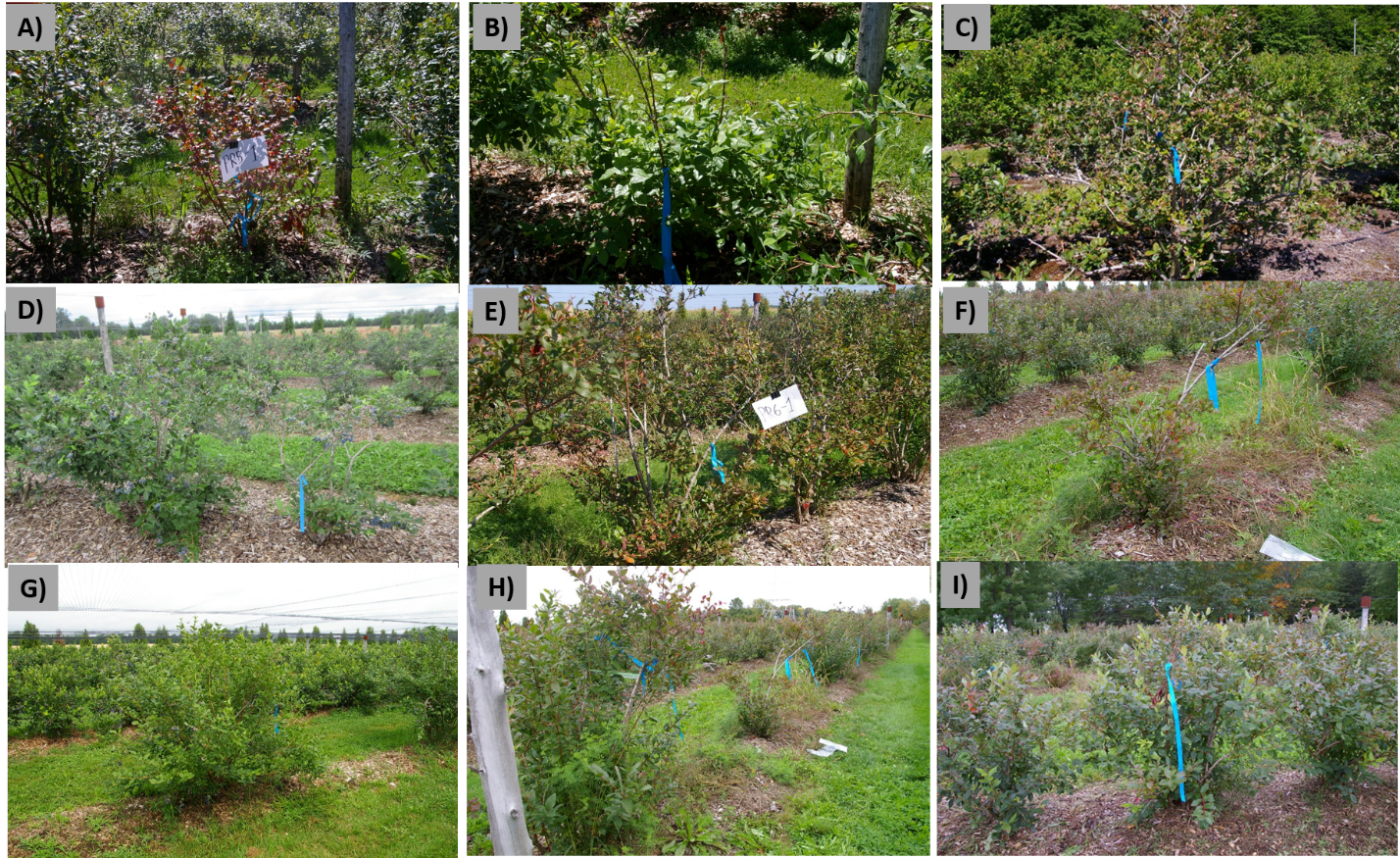


**Figure S2.** Selected phytoplasma-positive highbush blueberry plants: A) Qc4-PR5-1 (12 September 2017); B) Qc4-PR5-1 (19 June 2018); C) Qc8-P8 (11 June 2018); D) Qc4-PR6-1 (26 July 2016); E) Qc4-PR6-1 (12 September 2017); F) Qc4-PR6-1 (3 October 2018); G) Qc4-PR6-1 (8 August 2019); H) Qc4-PR6-1-A, centre, B (3 October 2018). Phytoplasma-negative plant : I) Qc4-PR6-1-C (3 October 2018).

| **Table S1.** Detection of BbSP phytoplasma DNA in LAMP-16S nested PCR discordant samples. These samples tested positive by LAMP but negative by nested PCR targeting the 16S rRNA-encoding gene. Results using qPCR targeting BbSP *cpn60* and nested PCR targeting BbSP ribosomal protein (*rp*) are indicated, along with the BLAST results obtained using the sequences of selected *rp* amplicons. | | | | | | | | | | | | | |
| --- | --- | --- | --- | --- | --- | --- | --- | --- | --- | --- | --- | --- | --- |
| **Sample** | **Collection date** | **tissue** | **colorimetric LAMP result** | **LAMP Tp, min** | **LAMP Ta, °C** | **LAMP result** | **16S nested** | **final call** | **Province** | **Site** | **C_q_** | ***rp* nested PCR** | ***rp* sequence BLAST result** |
| NS1P1 | 2016-08-02 | leaf | pos | 14.75 | 82.18 | pos | neg | pos | NS | NS1 | 37.23 | neg | ND |
| Qc4-PR6-1 | 2016-08-02 | fruit | pos | 12.75 | 82.29 | pos | neg | pos | QC | Qc4 | 24.02 | pos | ND |
| NS1P1 | 2016-09-13 | leaf | pos | 14.75 | 82.4 | pos | neg | pos | NS | NS1 | 25.35 | pos | ND |
| Qc1-P3B2 | 2016-07-19 | fruit | pos | 18 | 81.71 | pos | neg | pos | QC | Qc1 | 31.85 | pos | ND |
| NS6P2 OT | 2016-07-26 | leaf | pos | 21.25 | 81.88 | pos | neg | pos | NS | NS6 | 29.91 | pos | ND |
| Qc4-PR6-1 | 2016-07-19 | fruit | pos | 14.75 | 82.13 | pos | neg | pos | QC | Qc4 | 27.10 | pos | Blueberry stunt phytoplasma >98% |
| NS5P3 NT | 2016-07-26 | leaf | pos | 15.25 | 82.19 | pos | neg | pos | NS | NS5 | 28.78 | pos | Blueberry stunt phytoplasma >98% |
| NS6P1 OT | 2016-07-26 | leaf | pos | 15.25 | 82.18 | pos | neg | pos | NS | NS6 | 28.30 | pos | Blueberry stunt phytoplasma >98% |
| NS6P1 OB | 2016-07-26 | leaf | pos | 15.5 | 82.04 | pos | neg | pos | NS | NS6 | 28.25 | pos | ND |
| NS5P2 NB | 2016-07-26 | leaf | pos | 15.75 | 82.22 | pos | neg | pos | NS | NS5 | 35.93 | pos | Blueberry stunt phytoplasma >98% |
| NS6P2 NT | 2016-07-26 | leaf | pos | 15.75 | 82.23 | pos | neg | pos | NS | NS6 | 30.28 | pos | ND |
| NS5P4 NT | 2016-07-26 | leaf | pos | 16.25 | 82.37 | pos | neg | pos | NS | NS5 | 33.29 | pos | Blueberry stunt phytoplasma >98% |
| Qc1-P3C5 | 2016-07-19 | fruit | pos | 16.25 | 81.75 | pos | neg | pos | QC | Qc1 | 27.10 | pos | Blueberry stunt phytoplasma >98% |
| NS5P3 OB | 2016-07-26 | leaf | pos | 16.5 | 82.17 | pos | neg | pos | NS | NS5 | 27.63 | pos | Blueberry stunt phytoplasma >98% |
| NS5P3 OB | 2016-07-26 | leaf | pos | 17.75 | 82.51 | pos | neg | pos | NS | NS5 | neg | pos | Blueberry stunt phytoplasma >98% |
| NS1P4 | 2016-09-13 | leaf | pos | 15.75 | 82.2 | pos | neg | pos | NS | NS1 | 28.02 | pos | ND |
| NS6P2 OB | 2016-07-26 | leaf | pos | 15.75 | 82.13 | pos | neg | pos | NS | NS6 | 37.93 | pos | Blueberry stunt phytoplasma >98% |
| NS6P3 OT | 2016-07-26 | leaf | pos | 15.75 | 82.27 | pos | neg | pos | NS | NS6 | 29.81 | pos | Blueberry stunt phytoplasma >98% |
| NS6P4 NB | 2016-07-26 | leaf | pos | 15.75 | 82.08 | pos | neg | pos | NS | NS6 | 28.30 | pos | ND |
| NS6P4 NT | 2016-07-26 | leaf | pos | 15.75 | 82.31 | pos | neg | pos | NS | NS6 | 29.02 | pos | Blueberry stunt phytoplasma >98% |
| NS1P3 | 2016-08-02 | leaf | pos | 15.75 | 82.29 | pos | neg | pos | NS | NS1 | 30.50 | pos | Blueberry stunt phytoplasma >98% |
| NS6P4 OT | 2016-07-26 | leaf | pos | 16 | 82.08 | pos | neg | pos | NS | NS6 | 29.00 | pos | ND |
| NS6P3 NB | 2016-07-26 | leaf | pos | 16.5 | 82.08 | pos | neg | pos | NS | NS6 | 28.06 | pos | ND |
| NS1P2 | 2016-09-13 | leaf | pos | 17 | 82.5 | pos | neg | pos | NS | NS1 | 29.44 | pos | ND |
| NS1-P4 | 2016-08-16 | leaf | pos | 17 | 82.3 | pos | neg | pos | NS | NS1 | ND | pos | ND |
| NS1P4 | 2016-08-02 | leaf | pos | 17 | 82.23 | pos | neg | pos | NS | NS1 | 29.97 | pos | ND |
| NS1-P1 | 2016-08-16 | leaf | pos | 17.25 | 82 | pos | neg | pos | NS | NS1 | ND | pos | ND |
| NS7P3 OT | 2016-07-26 | leaf | pos | 18.25 | 82.22 | pos | neg | pos | NS | NS7 | 29.19 | pos | ND |
| NS7P3 OB | 2016-07-26 | leaf | pos | 19.25 | 82.27 | pos | neg | pos | NS | NS7 | 29.16 | pos | ND |
| NS6P3 NT | 2016-07-26 | leaf | pos | 20 | 81.98 | pos | neg | pos | NS | NS6 | 31.83 | pos | ND |
| ND, not determined | | | | | | | | | | | | | |

| **Table S2.** Sequences of amplification primers and hydrolysis probes used for detection and quantification of BbSP (‘*Candidatus* Phytoplasma asteris’) | | | | | |
| --- | --- | --- | --- | --- | --- |
| **Primer name** | **Sequence (5'-3')** | |  | **Amplification conditions** | |
|  |  | **Product size (bp)** | **Detection assay** | **qPCR** | **ddPCR** |
| D0528 | GATGGAGTTATTAATGTTGATG | 124 (BbSP)  107 (*Vaccinium* rubisco) | qRT-PCR (BbSP) and  ddPCR (BbSP and vacc-rubisco) | 1x 95°C, 3 min; 40 x 95°C, 10 sec; 58°C, 10 sec; 72°C, 30 sec (data collection) | 1x 94°C, 10 min; 50x 94°C, 30 sec; 55°C, 1:00 (ramp 2°C/sec); 1x 98°C, 10 min |
| D0529 | CTGTCATGCTTTCTCTATC |  |  |  |  |
| BbSP | FAM-AGAAGCGTA/ZEN/TCCTTTATCGTACTGC-IB |  |  |  |  |
|  |  |  |  |  |  |
| D0488 | CATCCAAGTTGAAAGAGATA |  |  |  |  |
| D0489 | AAACAGCTCTCCCATAG |  |  |  |  |
| vacc-rubisco | HEX-TCGTCCCTT/ZEN/GTTAGGATGTA-IB |  |  |  |  |
| SF1-F3 | ATTGATGCAGGAGCTAATCC | 242 | LAMP | 63°C 40 minutes  Anneal 90°C-75°C 0.05°C/sec (fluorescent assays only) | |
| SF1-B3 | AGGTACAATTTCTTGCACAGTA |  |  |  |  |
| SF1-FIP | ACTACCTGATGAAACAGCAGCC-GAGTTAGCTGCATTAACAGTTG |  |  |  |  |
| SF1-BIP | GCCCAAGCGATGCAAAAA-GTGGAGAAGCATATCCTTTATCGT |  |  |  |  |
| SF1-loopF | TTGAATATCTTCTTGGGCGTCT |  |  |  |  |
| SF1-loopB | GAAGTTGTTGAAGGATTGCAGT |  |  |  |  |

Table S3. Limit of detection (LOD) and limit of quantification (LOQ) for BBSP assays, as determined using an optimized curve-fitting model^1^.

| Assay | R.squared | Slope | Intercept | Low.95 | LOD | LOQ |
| --- | --- | --- | --- | --- | --- | --- |
| BBSP-qPCR^a^ | 0.997 | -3.411 | 39.07 | 100 | 7.53 | 149 |
| BBSP-LAMP^b^ | 0.820 | -2.439 | 22.60 | 170 | 60.24 | 249 |
| BBSP-LAMP^c^ | 0.710 | -2.543 | 17.41 | 0.771 | 0.466 | 6 |
| ^a^qPCR assay results calculated using copy numbers of BBSP *cpn60* plasmid – logarithmic function used: 2-parameter log-logistic | | | | | | |
| ^b^LAMP results calculated using ddPCR-determined *cpn60* copy number in naturally infected samples – logarithmic function used: 2-parameter Weibull type 2 | | | | | | |
| ^c^LAMP results calculated using ddPCR-determined fractional abundances in naturally infected samples – logarithmic function used: 2-parameter Weibull type 2 | | | | | | |

Table S4. Repeatability of the BBSP qPCR assay, determined using *cpn60* plasmid templates.

| Sample | *cpn60* copies | Mean C_q_ | Standard deviation | n | CV, % |
| --- | --- | --- | --- | --- | --- |
| Low | 5 | 36.68 | 1.112 | 11 | 3.03% |
| Medium | 100 | 32.17 | 0.420 | 12 | 1.31% |
| High | 10000 | 25.57 | 0.280 | 12 | 1.10% |

Table S5. Sensitivity and specificity of the LAMP assay, using nested PCR targeting the Aster Yellows *rp* locus^2^ as a gold standard. 95% CI = 95% confidence interval, calculated as described by Banoo et al.^3^

| Assay | **AY-rp nested PCR** | | | |
| --- | --- | --- | --- | --- |
| **LAMP** | **positive** | **negative** | **total** |  |
| **Positive** | 38 | 8 | 46 |  |
| **Negative** | 8 | 47 | 55 |  |
| **Total** | 48 | 198 | 101 |  |
|  | Value | 95%CI | Low | high |
| **test sensitivity** | 0.826 | 0.110 | 0.717 | 0.936 |
| **test specificity** | 0.855 | 0.093 | 0.761 | 0.948 |

Table S6. Sensitivity and specificity of the BBSP qPCR assay, using nested PCR targeting the Aster Yellows *rp* locus^2^ as a gold standard. 95% CI = 95% confidence interval, calculated as described by Banoo et al.^3^

| Assay | **AY-rp nested PCR** | | | |
| --- | --- | --- | --- | --- |
| **qPCR** | **positive** | **negative** | **total** |  |
| **Positive** | 39 | 8 | 47 |  |
| **Negative** | 5 | 47 | 52 |  |
| **Total** | 44 | 55 | 99 |  |
|  | Value | 95%CI | Low | high |
| **test sensitivity** | 0.886 | 0.094 | 0.793 | 0.980 |
| **test specificity** | 0.855 | 0.093 | 0.761 | 0.948 |

References

1 Klymus, K. E. *et al.* Reporting the limits of detection and quantification for environmental DNA assays. *Environmental DNA* **2**, 271-282, doi:<https://doi.org/10.1002/edn3.29> (2020).

2 Martini, M. *et al.* Ribosomal protein gene-based phylogeny for finer differentiation and classification of phytoplasmas. *Int. J. Syst. Evol. Microbiol.* **57**, 2037-2051 (2007).

3 Banoo, S. *et al.* Evaluation of diagnostic tests for infectious diseases: general principles. *Nat.Rev.Microbiol.* **4**, S21-S31 (2006).
